# Tailoring the Conductivity and Flexibility of Natural Poly(3-hydroxybutyrate-*co*-3-hydroxyvalerate)-Based Biocomposites by Introduction of Carbon Nanomaterials and Atactic Poly-3-hydroxybutyrate

**DOI:** 10.3390/ma18071585

**Published:** 2025-04-01

**Authors:** Viktoriia Talaniuk, Marcin Godzierz, Wanda Sikorska, Grażyna Adamus, Aleksander Forys, Urszula Szeluga

**Affiliations:** Centre of Polymer and Carbon Materials, Polish Academy of Sciences, Zabrze, M. Curie-Sklodowska Str. 34, 41-819 Zabrze, Poland; vtalaniuk@cmpw-pan.pl (V.T.); wsikorska@cmpw-pan.pl (W.S.); gadamus@cmpw-pan.pl (G.A.); uszeluga@cmpw-pan.pl (U.S.)

**Keywords:** biopolymer conductive composites, poly(3-hydroxybutyrate-*co*-3-hydroxyvalerate), atactic poly(3-hydroxybutyrate), multi-walled carbon nanotubes, graphene nanoplatelets, piezoresistive sensors

## Abstract

In the present work, we provide the development results of highly efficient conductive biopolymer composite films with potential use as piezoresistive sensors. Natural isotactic biopolymer poly(3-hydroxybutyrate-*co*-3-hydroxyvalerate) (PHBV) was selected as the primary biopolymer material. To reduce the crystallinity and improve the processability of PHBV, the synthetic atactic (R,S)poly-3-hydroxybutyrate ((R,S)-PHB) polyester was blended with the semicrystalline PHBV biopolyester. Graphene nanomaterials with different structures, comprising crude multi-walled carbon nanotubes (MWCNTs), oxidatively functionalized multi-walled carbon nanotubes (ox-MWCNTs) and graphene nanoplatelets (GNPs), were proposed as electroactive fillers. The preparation of the composites was based on a simplified solvent casting method and the conductive graphene fillers were dispersed into the biopolyester matrix without any further routines. As a result of the optimization, a PHBV/((R,S)-PHB) mass ratio of 70:30 was found to be the most promising composition to obtain composite films with the expected mechanical characteristics. The influence of graphene filler structure on the degree of crystallinity, viscoelastic, electrical, and piezoresistive properties obtained for of the composites was determined. The lowest PHBV/PHB matrix crystallinities of 37% (DSC) and 39% (XRD) were recorded for the composite with 1% ox-MWCNTs and 1% GNPs. The most promising piezoresistive responses were noted for composites filled simultaneously with 1% GNPs and 1% ox-MWCNTs or MWCNTs. However, a 1.5% deformation and recovery did not affect the initial conductivity of the PHBV/(R,S)-PHB +1%MWCNTs+1%GNP system (9 × 10^−5^ S/cm), while for the system with oxidized carbon nanotubes, the resistance increases by approximately 0.2% in relation to the initial value (8 × 10^−6^ S/cm).

## 1. Introduction

The basic problems of modern polymer composite engineering are to design and manufacture materials with superior mechanical strength, high thermal and electrical properties, while providing low weight and high resistance to external factors. However, the huge use for polymers in everyday living, in particular electronics, results in a number of environmental problems and it is recommended to use biomaterials in various fields of the economy. The materials that can meet these requirements are composites based on biopolymers, able to degrade relatively quickly in natural conditions, and filled with carbon nanomaterials, particularly in the area of eco-friendly electronics. Natural poly-3-hydroxybutyrate (PHB) is one of the biopolymers created by bacteria and its degradation by microbes in the environment is possible [1]. PHB, due to its high stability, non-cytotoxicity, non-immunogenicity and resistance to water, has found applications primarily as medical implants, surgical sutures, food packaging, disposable napkins, personal hygiene items, films, fibers, and hydrophobic coatings for paper and cardboard [2]. The good biocompatibility of PHB would makes it a promised material for biosensor technologies. Pure PHB as a highly crystalline material has to be additionally treated and modified, which unfortunately can affect the biodegradation process. Various modification procedures can be applied, including toughening based on the quenching and thermal treatment, compounding with both natural and synthesized polymers with proper molecular architectures, addition of natural fibers or rigid particulate fillers, and chemical functionalization [3,4,5,6]. In recent years, poly(3-hydroxyalkanoates) (PHA) blends have been extensively studied and recognized as an attractive polymeric material with significant future application potential. The interest in these materials stems from their desirable properties and wide range of possible applications. Synthetic analogs of PHAs can be obtained, for example, via anionic ring-opening polymerization (ROP) of β-substituted β-lactones. The simplest representative of this series is atactic poly[(R,S)-3-hydroxybutyrate], (R,S)-PHB, which can be obtained by ROP of racemic β-butyrolactone [7]. Atactic (R,S)-PHB has proven to be a valuable component in blends with other biodegradable polymers, such as PLA, PLGA or PHAs. Its blends with bacterial PHBV exhibit good miscibility in the molten state and solidify with a characteristic spherulitic morphology. These blends demonstrate interesting thermomechanical and processing properties, which can be adjusted by modifying their composition [8].

The properties of PHB can also be modified by graphene nanomaterials. Application of graphene structures allows the induction and tailoring for electrical conductivity by creating conductive networks of graphene particles in the polymer matrix. Electroactive conductive biopolymer-based composites are intensively studied for biomedical proposals, e.g., biosensors, and cells for tissue engineering, as well as modern electronics, i.e., internet of things, flexible electrodes, display devices, etc. The most common graphene materials used as polymer nanofillers, also biopolymers, are carbon nanotubes with different aspect ratios, graphene oxide (GO) by oxidation of graphite, reduced graphene oxide (rGO), graphene nanoplatelets and others, homogenously distributed in the polymer matrix [7]. The structure, surface characteristic and physicochemical features of these fillers and interactions with the polymers significantly influence the microstructure and enhance electrochemical characteristics and thermal properties of nanocomposites, crucial for potential applications [9,10,11].

A study performed by Mahamud et al. [12] on the effect of GNP content (0–5 wt.%) on electrical conductivity of PHBV-based biocomposites formulated by the solvent casting indicated that a filler content at 5 wt.% induced the best electrical conductivity to be 3.83 × 10^−3^ S/cm of the composite characterized by higher crystalline regions. The poly-3-hydroxybutyrate-based composites with graphene nanoplatelets at concentrations in the range from 0 to 30 wt.% showed that the highest GNP concentration and hot-pressing processing [13] resulted in a very low film resistance of 6 Ω sq^−1^, which makes PHBV/GNP composites a promising candidate for green flexible electronics. Piao at al. [14] proved that pristine single-walled carbon nanotubes (SWCNTs) dispersed in a polymer matrix contributed to a p-type thermoelectric material, while SWCNTs chemically functionalized with polyethyleneimine created an n-type thermoelectric composite dedicated for power generation.

Amaral Montanheiroa et al. [15] investigated the influence of functionalized MWCNTs on the electrical properties of PHBV nanocomposites containing 0.5 wt.% MWCNTs. Nanocomposites with initial MWCNTs showed an electrical conductivity of 1.2 × 10^−5^ S cm^−1^; for those with MWCNT-COOH, the conductivity was reduced to 1.0 × 10^−6^ S cm^−1^; and for those with MWCNT-OH, the conductivity value was lowered to 3.0 × 10^−6^ S cm^−1^. The initial MWCNTs have an ideal structure, with no defects; therefore, the transport of charge is favored. The functionalization resulted in some defects on the MWCNT surface and the electrical transport process is changed compared to the pristine nanotubes with an almost perfect structure. The increase in electrical conductivity was studied for various biopolymer composites. Yang et al. reported [16] the change in the electrical resistivity from approximately 1 × 10^12^ Ω/sq to 1 × 10^2^ Ω/sq, when the MWCNT contents ranging from 0 to 8 wt.%.

Combining GNPs or graphene oxide with carbon nanotubes in biopolymer nanocomposites can results in synergistic improvements in electrical parameters and additionally in thermal conductivity. Dos Anjos at al. [17] explored the effect of GNPs and MWCNTs on improving the electrical and electromagnetic characteristics of poly(lactic acid) and poly(3-hydroxybutyrate-*co*-3-hydroxyvalerate) blends. Nanocomposites obtained by technique of injection molding and extrusion, containing 1 wt.% MWCNTs and 3 wt.% GNPs were characterized by conductivity above 10^−4^ S/cm, and this value allows to be used as an antistatic packaging. Cataldi et al. [18] studied polyhydroxyalkanoate (PHA) biopolyester composites blended in melt with GNPs and a composition of GNPs and carbon nanofibers. The nanocomposites with GNPs with carbon nanofibers showed a percolation threshold of electrical conductivity at a lower content of nanofiller compared with the PHA system with GNPs. The electrical conductivity for 15 wt.% filler content was approximately 6 times better than for the nanocomposite with GNPs.

The PHB composites with nanostructured materials offer opportunity to obtain sensors, especially piezoresistive and temperature sensors with suitable sensitivity, stability, environmental compatibility, reduced power consumption and a low coefficient of heat dissipation. Piezoresistive sensors based on biopolymers with graphene fillers offer advantages such as an uncomplicated design and simple preparation methods through direct addition of conductive fillers to polymers. Moshkriz et al. [19] developed new highly flexible and very sensitive strain sensors for biomedical applications based on a thermoplastic vulcanizate mixture of silicone rubber and PHBV with silicon-modified graphene oxide as a filler. The sensors were characterized by a considerably low percolation threshold of approximately 1.3 vol.%. The composites showed high stretchability, sensitivity (GF = 375), and an ability to stretch beyond 100% of the applied strain. Research on PHBV systems modified with poly(butylene adipate-*co*-terephthalate) (PBAT) showed that MWCNTs and GNPs, applied both as individual filler and simultaneously, reduced the electrical resistance of composites by creating conductive pathways of these graphene nanofillers in the polymer matrix [20].

However, it should be emphasized that the introduction of graphene structures which induce the electrical conductivity of PHB increases its crystallinity [21]. Shan et al. [22] reported the influence of MWCNTs on the degree of crystallization of PHBV, and the data proved that nanotubes addition induced an increase in crystallinity degree and size of crystallite sizes as a result of nucleation phenomena. Lemes at al. [23] in their work studied the influence of MWCNT content (0.05–2 wt.%) on morphology, thermal and mechanical properties of PHBV nanocomposites produced by solvent casting. The higher MWCNT concentration in nanocomposites caused an increase in crystallinity degree, from 56.1% for PHBV, to 69.6% for the nanocomposite filled with 2 wt.% of MWCNTs. Montanheiro et al. for PHBV nanocomposites with carbon nanotubes pre-dispersion in acetone reported decreased crystallinity from 62% for pure PHBV to 55% for PHBV composite with 0.5 wt.% MWCNTs [24].

In this work, our objective was to develop a highly effective, environmentally safe and economically profitable piezoresistive biosensors, specifically designed to optimal cyclic work. The application of electrically conductive graphene nanomaterials resulted in an increase in the crystallinity degree of final PHBV-based composite films; therefore, the modification of isotactic PHBV with active poly-3-hydroxybutyrate was optimized to improve composite film formation ability. The microstructure of the polymer chain significantly influences the physical characteristics of polymer. Atactic (R,S)-PHB is an amorphous polymer that exhibits elastomeric properties at room temperature, whereas isotactic PHBV is a highly crystalline material with a high melting point. Blends of PHBV and (R,S)-PHB show reduced crystallinity and possess unique thermomechanical and processing properties that can be adjusted by modifying the blend composition [25]. In the study, a PHBV/(R,S)-PHB blend with a 70:30 wt.% was identified as the optimal polymer matrix, offering suitable properties for film formation with graphene-based materials.

The sensing properties of composite materials are sensitive to the structure and concentration of electroactive graphene nanomaterials The deformation of 1.5% and recovery did not disturb the conductive pathways of nanofiller particles and consequently did not change the conductivity of the composite with 1% ox-MWCNTs+1%GNPs.

## 2. The Experimental Section

### 2.1. Materials

Poly(3-hydroxybutyrate-*co*-3-hydroxyvalerate) (PHBV), manufactured by Tianan Biologic Material Co (tradename ENMAT Y1000, Ningbo, China) with 1.2% of HV units, was used as a biopolymer matrix (commercial material). As a modifier of PHBV, poly[(R,S)-3-hydroxybutyrate] was proposed. The (R,S)-β-butyrolactone (β-BL by Sigma–Aldrich, Steinheim, Germany) was used as a monomer to the synthesis of poly[(R, S)-3-hydroxybutyrate] ((R,S)-PHB) and was purified before use by distillation over CaH_2_. Tetrabutylammonium acetate (AcNBu4) was supplied by Sigma–Aldrich Chemie GmbH, Steinheim, Germany and dried under vacuum at 45 °C for 6 h before used. Chloroform, n-hexane, and hydrochloric acid (36%) (Avantor Performance Materials, Gliwice, Poland) were used with no further purification.

To introduce the required electrical conductivity and to provide the optimized mechanical and thermal performances of the PHBV/(R,S)-PHB matrix, highly conductive graphene nanomaterials with different structures were used as active nanofillers in the composite preparation procedure. Commercially available multi-walled carbon nanotubes (MWCNTs) (C ≥ 95%, with an average length of 5 μm and an average diameter of 6–9 nm), were sourced from Sigma-Aldrich. GNPs (xGnP^®^ M-15, C ≥ 95%, with an average XY dimension of 15 μm, a thickness of 6–8 nm, and a specific surface area of 120–150 m^2^/g) provided by Sigma-Aldrich were selected as a 2D graphene conductive material.

### 2.2. Procedures

#### 2.2.1. Oxidation of MWCNTs

Oxidized MWCNTs were prepared using concentrated nitric acid (conc. 69%, Chem-Pur, Poland). MWCNTs prepared in this way were kept in boiling concentrated HNO_3_ under a reflux condenser for approximately 50 h at 120 °C to completely remove amorphous carbon, catalyst traces and their carriers, and oxidize the graphene surfaces. Oxidized nanotubes (ox-MWCNTs) were washed with deionized water, followed by NH_4_OH, water solution, HCl solution and once again with deionized water to stabilize the pH of filtrate.

#### 2.2.2. The Synthesis of Poly[(R,S)-3-hydroxybutyrate], (R,S)-PHB

The (R,S)-PHB was synthesized by β–BL ring-opening anionic polymerization initialized with tetrabutylammonium acetate, using the original method reported previously [7]. The polymerization was carried out at room temperature in bulk and its progress was monitored by Fourier transform infrared spectroscopy (FT-IR) from changes in the intensity of the peak characteristic of the carbonyl groups in the β–BL monomer and the resulting (R,S)-PHB polyester at 1815 cm^−1^ and 1735 cm^−1^, respectively. The (R,S)-PHB polyester solution in chloroform was prepared and then was protonated by an equivalent amount of 1% aqueous HCl solution. The precipitation of the resulting product was performed in cold n-hexane and dried to constant weight under vacuum at room temperature.

#### 2.2.3. The Preparation of Thin Films of Biopolymer Composites

**Figure 1:**
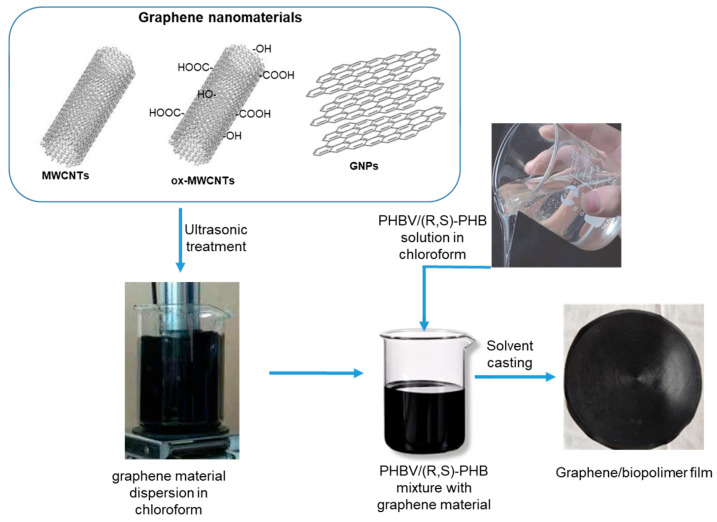
The preparation of films based on the PHBV/(R,S)-PHB and graphene nanomaterials.

To investigate the effect of the structure and content of graphene fillers on the morphology, crystallinity degree and dynamic mechanical, electrical conductive and sensing properties, thin composite films based on the PHBV/(R,S)-PHB matrix and graphene materials were prepared. The optimized composition of PHBV/(R,S)-PHB polymer components was set to 70:30 wt.%, and both components were individually dissolved in 5 mL of chloroform. At the same time, graphene materials, i.e., carbon nanotubes, oxidized carbon nanotubes, and/or GNPs were ultrasonically treated in chloroform. For the preparation of the composites, individual fillers were used at 2 wt.%. In addition, composites with a mixture of 1 wt.% raw or oxidized MWCNTs and 1 wt.% GNPs were prepared. The obtained solutions of the polymer mixture and the corresponding carbon nanomaterial were then compounded and homogenized by mechanical stirring (Figure 1). The solution casting method was finally used to prepare thin, free-standing films with favorable mechanical properties. Composite films were prepared at 19 °C and 45% humidity.

### 2.3. Characterization

#### 2.3.1. X-Ray Diffraction

The X-Ray Diffraction analysis of graphene nanomaterials and graphene/biopolymer composites were conducted using a diffractometer D8 Advance (Bruker Corporation, Billerica, MA, USA). As a X-ray source, the diffractometer used a copper (Cu) Kα cathode with a wavelength (λ) of 1.54 Å (angstroms).

The scan rate was set at 0.6°/min, the scanning step was set to 0.02° and the scanning range was from 5° to 90° 2Θ. The measurements were performed using Bragg–Brentano geometry, a common setup for powder XRD analysis. The DIFFRAC.EVA software (ver. 5.1) was used in the identification of the phases analyzed. Within this software, the ICDD (International Centre for Diffraction Data) PDF#2 (Powder Diffraction File) database was used. The crystallinity of biopolymer phases was calculated using method of the peak decomposition method. All measurements and analyses were performed three times to ensure statistically reproducible results.

#### 2.3.2. Scanning Electron Microscopy

The structure and surface morphology of the graphene nanomaterials and brittle fracture area of graphene/biopolymer composites, filler particles distribution in the polymer, and the thickness of composite films were examined using scanning electron microscopy (SEM, FEI Quanta 250 FEG equipped with a secondary electron detector (Thermo Scientific, Waltham, MA, USA). SEM images of dried powder of graphene materials mounted on carbon tape were registered using 5 kV of accelerating voltage. SEM observations of composite samples was performed using the low vacuum mode and accelerating voltage of 5 kV.

#### 2.3.3. Atomic Force Microscopy (AFM)

AFM measurements were performed using a Dimension ICON AFM microscope equipped with a NanoScope V controller (BRUKER Corporation, Santa Barbara, CA, USA) operating in the soft tapping mode in an air atmosphere with standard 125 μm single-crystal-doped silicon cantilevers with flexural stiffness of 42 N/m (Model PPP-NCH-10, NANOSENSORS, Neuchatel, Switzerland). The images were captured with a piezoelectric scanner featuring with a nominal dimensions of 85 × 85 μm. Micrographs were acquired using NanoScope Analysis 1.9 software (BRUKER Corporation, Santa Barbara, CA, USA).

#### 2.3.4. Cryogenic Transmission Electron Microscopy (Cryo-TEM) Measurements

Cryogenic Transmission Electron Microscopy (cryo-TEM) imaging was performed using a Tecnai F20 X TWIN microscope (FEI Company, Hillsboro, OR, USA) which operates using a field emission gun at an acceleration voltage of 200 kV. Images were collected by a Gatan Rio 16 CMOS 4k camera (Gatan Inc., Pleasanton, CA, USA) and then processed with Gatan Microscopy Suite (GMS) software version 3.31.2360.0 (Gatan Inc., Pleasanton, CA, USA). Samples were prepared by vitrification of aqueous solutions placed on cooper grids covered with a carbon film (Quantifoil R 2/2; Quantifoil Micro Tools GmbH, Großlöbichau, Germany). Prior to use, the grids were activated for 15 s with an oxygen plasma using a Femto plasma cleaner (Diener Electronic, Ebhausen, Germany). Cryogenic samples were prepared by applying a drop (3 μL) of the suspension to the grid, then blotting with filter papers and immediately plunged in liquid ethane using a fully automated Vitrobot Mark IV desiccant device (Thermo Fisher Scientific, Waltham, MA, USA). The vitrified specimens were stored in liquid nitrogen until placed in a cryo-TEM holder (Gatan 626; Gatan Inc., Pleasanton, CA, USA) and analyzed at −178 °C in the TEM.

#### 2.3.5. Differential Scanning Calorimetry

The melting and crystallization parameters of the PHBV/(R,S)-PHB mixture and its composites with graphene nanomaterials were evaluated using a differential scanning calorimeter (DSC Q2000, TA Instruments, New Castle, DE, USA). DSC runs were conducted in a nitrogen atmosphere at a nitrogen flow rate of 50 mL/min through the measuring chamber. A sample of approx. 10 mg was placed in standard aluminum nonhermetic pans and the temperature was raised at a rate of 10 °C/min from −50 °C to 200 °C. The melted samples were held in 200 °C for 5 min to minimize the effect of the sample thermal history. Subsequent cooling to room temperature was performed at a rate of 10 °C/min. From the data obtained in this run, the temperature (*T*_c_) and the enthalpy (Δ*H*_c_) of crystallization were determined. Following the first melting and crystallization measurements combined, the samples were reheated in a further run from 0 °C to 200 °C. According to ISO 11357-3 standard [26], the temperature (*T*_m_) and enthalpy (Δ*H*_m_) of the melting process were determined. Additionally, the temperature of glass transition (T_g_) for the amorphous phase of the polymer was taken.

The crystallinity degree (*X_c_* (%)) of isotactic poly-3 hydroxybutyrate of both pure PHBV/(R,S)-PHB and composites with graphene nanomaterials was calculated using Equation (1):

(1) $$X_{c}\left(\%\right)=\frac{{∆H}_{m}}{{∆H}_{m}^{0}\varphi }\cdot 100$$

where Δ*H_m_* (J/g) is the enthalpy of melting enthalpy from the second reheating cycle obtained by integrating the surface under the melting peak, *φ* is polymer weight fraction, the Δ*H*^0^*_m_* is the enthalpy of melting enthalpy of a completely (100%) crystalline PHBV (theoretical value of 146 J/g) [27,28,29]. However, some literature reports present theoretical value of melting enthalpy of 100% crystalline PHBV equal 109 J/g [21,23]. In the calculation of the degree of the crystallinity of the composites studied the value of d Δ*H*^0^*_m_* 146 J/g was used.

#### 2.3.6. Dynamic Mechanical Analysis

Dynamic Mechanical Analysis (DMA) was carried out using a DMA analyzer (DMA 2980, TA Instruments, New Castle, DE, USA). A film tension clamp was used for thin film samples with a thickness ranging from 0.1 to 0.25 mm, a width of 6–7 mm, and a length of 7–10 mm.

The samples were subjected to oscillations at a frequency of 1 Hz. A 10 µm oscillation amplitude was used. Measurements were performed with a 0.01 N static force and 120% of autostrain applied to the sample. The storage modulus (*E*′) and loss modulus (*E*′′) were determined from −60 °C to 100 °C at a temperature increase rate of 3 °C/min. The T_g_ was identified as the temperature of the loss modulus (*E*′′) peak. However, in many practical applications, the peak temperature of the loss factor (tan δ) is often considered as the T_g_.

#### 2.3.7. Electrical and Piezoresistive Characterization

Measurements of conductivity and piezoresistive properties of PHBV/(R,S)-PHB-based composites with graphene fillers were carried out. The samples for conductivity measurements were approx. 20 × 5 mm in size. High-purity silver paste (SPI Supplies, 05002-AB, West Chester, PA, USA) was used to cover opposite sides of samples with a spacing of approximately 10 mm between them. The copper wires have then been connected to the electrodes using the silver paste. The composite resistance was measured with a RMS multimeter (UNI-T, UT804, Uni-Trend Technology Co., Dongguan, China). Measurements were carried out for not less than three different samples.

The conductivity (*σ*) values were calculated using Equation (2):

(2) $$\sigma =\frac{L}{R\cdot S}$$

where *σ* is conductivity (S/cm), *L* is distance between electrodes in cm, *R* is sample resistance in Ω, and *S*—cross-section in cm^2^.

To determine the piezoresistive characteristics of the composites studied, first silver electrodes were coated with a silver paste of high purity on both sides of the sample and then copper wires were fixed to the electrodes. Electrodes were coated with a silver paste of high purity on both sides of the sample, and then copper wires were attached to the electrodes. Changes in resistance values as a function of deformation were observed using a Keithley 6485 picoammeter (Keithley Instruments, Cleveland, OH, USA) integrated with LabVIEW 2013 software. A 1 V initial voltage was applied using an external power supplier. The tensile deformation was conducted using a DEBEN microtensile machine with a 2 kN cross-head and moving at a speed of 0.4 mm/min. In current studies, deformations of 1.5% and 3% were studied [30,31,32]. The piezoresistive responses have been registered in room temperature.

## 3. Results and Discussion

### 3.1. Characterization of Polymer and Graphene Materials Used in Composites

**Figure 2:**
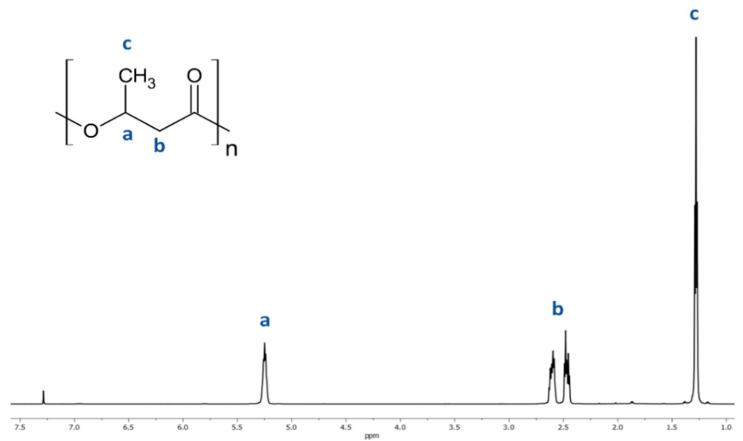
Spectrum of ^1^H NMR of poly((R,S)-3-hydroxybutyrate).

The resulting atactic (R,S)-PHB polyester was analyzed using ^1^H-NMR. The presence of characteristic peaks was correlated to the protons of the 3-hydroxybutyrate repeating units using 1H NMR analysis of (R,S)-PHB; see the spectrum in Figure 2. Signals located at 1.28 (c); 2.50 (b) and 5.26 (a) ppm and correspond to the protons of the methyl, methylene, and methine groups of 3-hydroxybutyrate repeat units of (R,S)-PHB polyester, respectively.

The structure and properties of the surface of both untreated and oxidized MWCNTs and GNPs used as fillers in the PHBV/(R,S)-PHB blend were analyzed by TEM and SEM imaging. Microscopic photographs of these graphene-based materials are presented in earlier work [20]. The Raman spectroscopy results were used to assess the structural organization of the graphene layers in the fillers. This technique identified two characteristic peaks in the Raman spectrum: the G band, which characterizes graphite-like materials with a peak located at approximately 1600 cm^−1^, and a D band, associated with structural defects found for carbon nanotubes, appearing at approximately 1350 cm^−1^ [20]. Additionally, the spectrum revealed a band at 2670 cm^−1^, which corresponds to the first G-mode overtone, and another at 2870 cm^−1^, representing the first D-mode overtone. The degree of structural defects in graphene materials was evaluated by the ratio of D to G peak intensities (I^D^/I^G^). The higher values of I^D^/I^G^ observed for MWCNTs and ox-MWCNTs compared to GNPs indicate the presence of more structural irregularities in the nanotubes. These defects may result not only from irregularities such as pores and impurities in the graphene layers, but also from sp^3^ hybridization due to oxygen groups.

### 3.2. Scanning Electron Microscopy c (SEM) Characterization of the PHBV/(R,S)-PHB Blend

**Figure 3:**
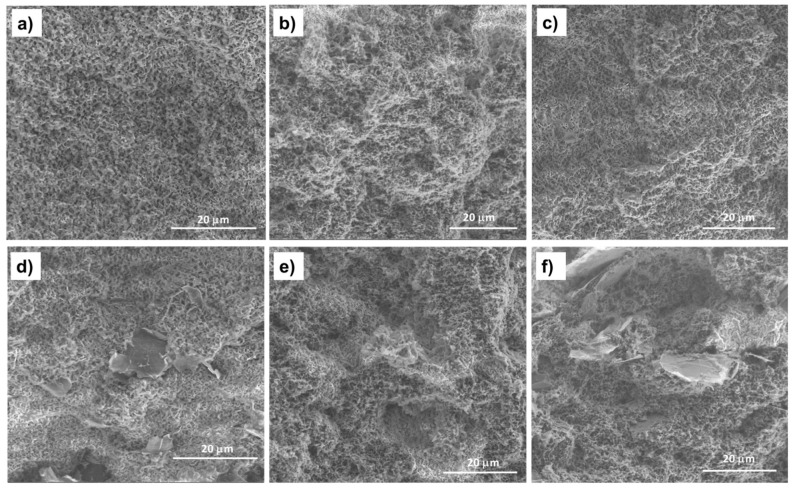
SEM images of the fracture surfaces of the PHBV/((R,S)-PHB) blend film (**a**) and its composites: (**b**) with 2 wt.% MWCNTs, (**c**) with 2 wt.% of ox-MWCNTs, (**d**) with 2 wt.% GNPs, (**e**) with 1 wt.% MWCNTs and 1 wt.% GNPs, and (**f**) with 1 wt.% ox-MWCNTs and 1 wt.% GNPs.

The morphology of PHBV/(R,S)-PHB polymer blends can arise from factors such as the chemical structure and polymer component ratio, their physicochemical parameters, mainly viscosity, the nature of the interfacial surface, and processing conditions such as time, temperature and pressure [33]. To gain a deeper understanding of the PHBV/(R,S)-PHB microstructure, both in pure form and filled with graphene nanomaterials, were carried out using SEM analysis, which provided a qualitative assessment of the composite phase morphology. The summary of SEM images of the PHBV/(R,S)-PHB blend and composites films in Figure 3 enables insights into the microstructure of the polymer phase, boundary areas between filler and polymer and the dispersion quality of MWCNTs, ox-MWCNTs and GNPs in the PHBV/((R,S)-PHB blend.

The fracture surface morphology of the PHBV/(R,S)-PHB blend differs significantly from that of the pure PHBV film reported in the literature [20], for which a smooth and flat surface can be observed. The cross-section of the PHBV/(R,S)-PHB film contains nanopores formed as a result of the immiscibility of crystalline PHBV and amorphous (R,S)-PHB. The resulting structure of the PHBV/(R,S)-PHB) composite films is similar to the structure of the polymer matrix, and the pore sizes are comparable. The morphology of the PHBV/(R,S)-PHB matrix (Figure 3a) remains unchanged after the introduction of 2 wt.% MWCNTs, both initial (Figure 3b) and oxidized (Figure 3c), GNPs (Figure 3d), and in combination with MWCNTs (Figure 3e,f).

In the nanocomposites with 2 wt.% GNPs used individually and 1% GNPs used in combination with 1% MWCNTs or with ox-MWCNTs, areas with relatively well-separated and uniformly dispersed graphene layers in the PHBV/(R,S)-PHB blend can be observed. In the case of the composite with 2 wt.% raw and oxidized MWCNTs, the nanotubes are wrapped and covered by the polymer phase and it is not possible to identify them on the SEM microphotographs.

### 3.3. AFM Characterization

Analysis of the fracture surface topology of pure PHBV/(R,S)-PHB blend film and its composite films was performed by atomic force microscopy using the soft tapping mode.

**Figure 4:**
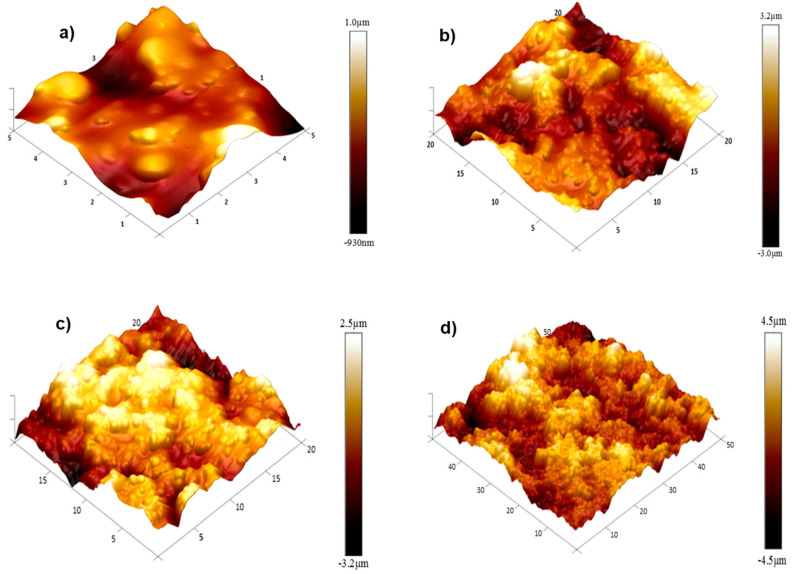
AFM 3D images of the fracture surfaces of the PHBV/((R,S)-PHB) polymeric blend film and its composite films: (**a**) with 2 wt.% MWCNTs, (**b**) with 2 wt.% of ox-MWCNTs, (**c**) with 2 wt.% GNPs, and (**d**) with 1 wt.% CNTs and 1 wt.% GNPs.

Figure 4 shows the 3D image of the fracture surface of pure PHBV/(R,S)-PHB shows a smooth and uniform structure with visible fracture marks indicating the brittle nature of the material. Some microcracks were observed, which characterize the fractography of the pure polymer without additives.

A significant strengthening of the fracture surface is seen for the PHBV/(R,S)-PHB (Figure 4a) composite with 2 wt.% MWCNTs (Figure 4b), which leads to a reduced number of cracks and an increase in the irregularity. Carbon nanotubes contribute to a more complex surface morphology, with increased stiffness and energy intensity. The PHBV/(R,S)-PHB composite with 2 wt.% oxidized MWCNTs (Figure 4c) shows an even more pronounced texture on the fracture surface Oxidation of MWCNTs leads to better adhesion with the polymer, which contributes to a finer but more rigid texture.

The fracture surface of the composite containing 2 wt.% GNP is characterized by a large number of layered structures and wave-like formations. The GNP form a significant number of barriers, which increase the mechanical stiffness of the composite and give it greater resistance to fracture. The composite material with 1 wt.% MWCNTs and 1 wt.% GNPs (Figure 4d) shows a complex multilayered texture, with pronounced distinct morphology at the fracture sites. This combination reduces the number of microcracks by combining the reinforcing properties of both types of fillers, creating a synergistic effect and increasing the strength of the material.

### 3.4. Cryo-TEM Characterization

Cryo-TEM characterization was performed for the PHBV/(R,S)-PHB (70:30) blend and for pure individual constituents, i.e., natural isotactic PHBV and atactic synthetic (R,S)-PHB.

**Figure 5:**
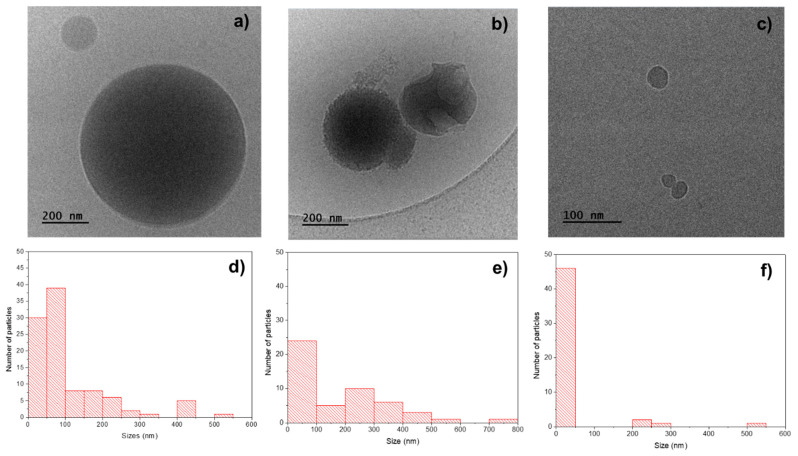
Cryo-TEM images of the fracture surfaces of (**a**) pure PHBV/(R,S)-PHB, (**b**) PHBV, and (**c**) (R,S)-PHB and histograms with particles size of distribution in (**d**) PHBV/(R,S)-PHB, (**e**) PHBV, and (**f**) PHBV/(R,S)-PHB.

The cryo-TEM image of the PHBV/(R,S)-PHB specimen (Figure 5a) shows spherical particles with sizes: 15–550 nm. The average size (measured for 100 objects) is 110 nm and the size distribution measured for 100 particles is highlighted in the histogram in Figure 5d.

The cryo-TEM image of pure PHBV (Figure 5b) shows spherical particles with sizes of 10–800 nm, prevailing particles with sizes of 10–80 nm which tend to form aggregates. The average dimension of the particles of PHBV (measured for 50 objects) is 110 nm and the detailed size distribution measured for 50 objects is presented in the histogram in Figure 5e. For pure (R,S)-PHB, similarly, the spherical particles were noticed—however, with smaller dimensions of 15–50 nm. Only a small number of particles with sizes of 200–550 nm were noted. The average size (measured for 50 objects) is 45 nm and the size distribution measured for 100 particles is shown in the histogram in Figure 5f.

**Figure 6:**
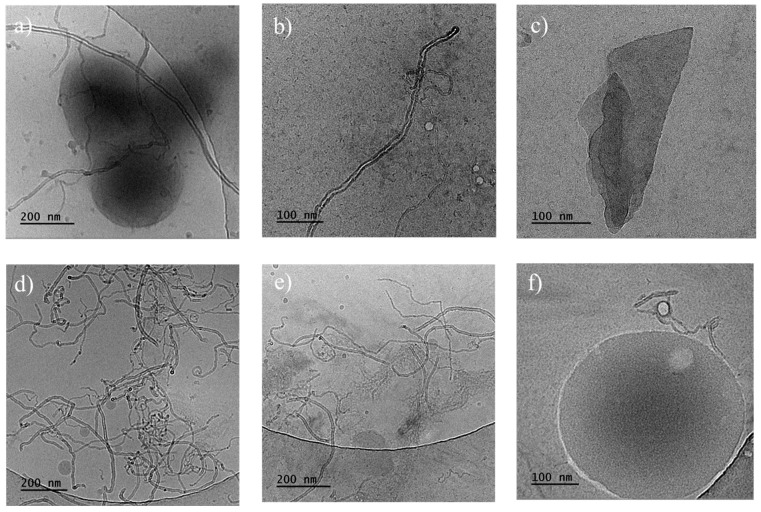
Cryo-TEM images of the fracture surfaces of PHBV/(R,S)-PHB composites: (**a**–**c**) with 1 wt.% MWCNTs and 1 wt.% GNPs; (**d**–**f**) with 1 wt.% ox-MWCNTs and 1 wt.% GNPs.

Cryo-TEM measurements of the PHBV/(R,S)-PHB composite with 1 wt.% MWCNTs and 1 wt.% GNPs revealed that multi-walled carbon nanotubes exhibit an elongated morphology, predominantly arranged in a random manner (Figure 6). The boundaries between the poly(3-hydroxybutyrate)-based matrix and the carbon fillers can be clearly distinguished. Spherical particles with sizes of 15–50 nm were observed separately. Small aggregates of graphene flakes can be observed within certain areas, which could influence the composite properties.

Cryo-TEM images for the slice of the PHBV/(R,S)-PHB composite with 1 wt.% ox-MWCNTs and 1 wt.% GNPs indicated that multi-walled carbon nanotubes also exhibit an elongated type of morphology, mostly arranged in a random way with entanglement sites. Spherical particles with sizes of 15–220 nm were observed separately or adjacent to carbon nanotubes.

### 3.5. The Characterization and Crystallinity of the Biopolymer Matrix by XRD and DSC

**Figure 7:**
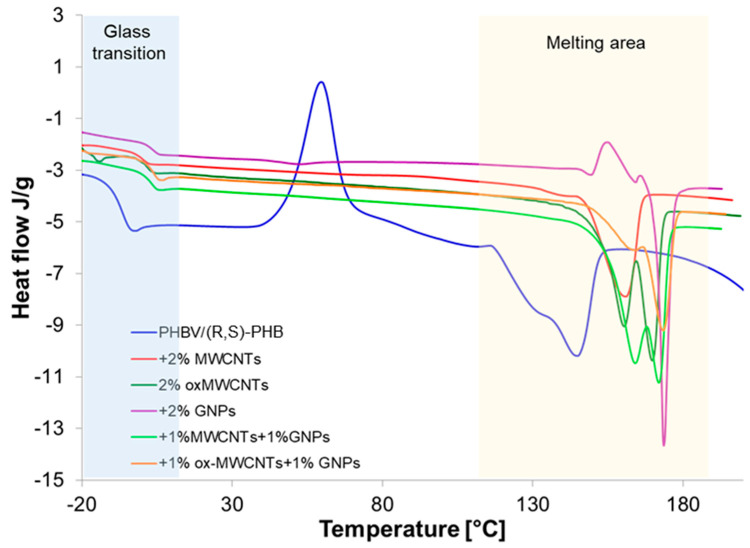
Glass transition and melting endotherms of PHBV/(R,S)-PHB composition and PHBV/(R,S)-PHB composites with MWCNTs, ox-MWCNTs and GNPs taken as third DSC runs.

The investigation of glass and melting transitions of the PHBV/(R,S)-PHB blend in composites with different graphene nanofillers can supply important information on how the structure of graphene material affects the crystalline nature of polymer phases, composite morphology, interactions between polymer and graphene fillers, and macroscopic properties of the composites studied. This can supply important information on how the structure of graphene materials affects the crystalline nature of polymer phases. To evaluate these relationships, the PHBV/(R,S)-PHB film and its composites were analyzed using DSC non-isothermal analysis. The T_g_ and melting parameters were determined based on the third runs. The results of this study are shown in Figure 7.

Two melting points are observed for pure PHBV/(R,S)-PHB) composition and all composites studied. The presence of two peaks can be a result of the melting of PHBV crystals characterized by different structures and lamellar thicknesses [34]. The peak at lower temperature is probably the peak of the melting of pre-formed crystals, whereas the peak at a higher temperature is attributed to the melting process of crystals that underwent recrystallization. Double melting peaks for composites with graphene materials can be additionally explained as an effect of graphene fillers as nucleating agents on PHBV melting behavior [35]. However, the profile and temperature locations of these peaks depend on the graphene materials applied to prepare these composites. The most critical could be the relatively rapid heating required to overcome the process of reorganization, which appears to be quite rapid in these samples. A temperature ramp rate of 10°/min in DSC cycles can be too low.

The melting parameters of the PHBV/(R,S)-PHB mixture and its composites, i.e., melting temperature (*T*_m_), melting enthalpy value (Δ*H*_m_) and glass transition of amorphous poly-3-hydroxybutyrate determined from third DSC run curves are summarized in Table 1. For comparison, data are also presented for pure PHBV. The T_m_ and crystallinity degree are similar to the literature data on DSC studies for PHB [36]. The DSC curve of the PHBV/(R,S)-PHB system is characterized by the presence of an exotherm corresponding to cold crystallization of poly-3-hydroxybutyrate occurred above the T_g_ of PHBV, suggesting the sufficient mobility of the chains in the crystallization process. This phenomenon is not observed for the composites.

The temperature of glass transition of the polymer phase for all composites studied is higher than that of the unfilled initial PHBV/(R,S)-PHB composition and is due to the limited mobility of the polymer chains and graphene filler particle–polymer interactions. The highest T_g_ was recorded for composites when both 1D MWCTs and 2D GNPs were applied. The DSC melting results observed indicate that the fillers used similarly like in the case of glass transition constrain the movement of the segmental poly-3-hydroxybutyrate due to filler–polymer interactions. The incorporation of raw MWCNTs results only in a minor increase in melting temperatures of PHBV by several degrees Celsius. The most significant increase is observed with the simultaneous addition of both 1% MWCNTs and 1% GNP and 1% ox-MWCNTs and 1% GNPs. The melting enthalpy of PHBV in composites based on PHBV/(R,S)-PHB also increases with the addition of fillers in comparison with the PHBV/(R,S)-PHB The highest Δ*H*_m_ was noted for the composite with 2% ox-MWCNTs, while the lowest value for the composite +1% ox-MWCNTs+1%GNPs.

The crystallinity degree of PHBV/(R,S)-PHB composites with graphene fillers, established by XRD method, is also given in Table 1. The obtained tendency is consistent with the data determined using DSC, but with lower crystalline fraction values.

Crystallinity is observed to be highest in the film of natural PHBV. The mixture PHBV with (R,S) has a significantly lower overall crystallinity of the system, whereas the introduction of carbon nanofillers causes an increase in comparison with the PHBV/(R,S)-PHB blend. The crystallinity value of 39% found for composite with 1% ox-MWCNTs and 1% GNPs is the lowest value among the composites studied.

### 3.6. Dynamic Mechanical Studies

**Figure 8:**
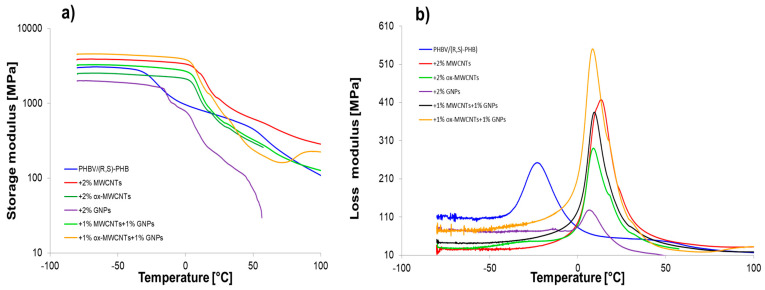
The storage modulus (log scale) (**a**) and loss modulus (**b**) as functions of temperature for PHBV/(R,S)-PHB and composites.

Graphene nanomaterials with different structures were introduced into polymer systems to induce electrical conductivity. Additionally, when biopolymers are used in composites, it is necessary to ensure that they have sufficient mechanical performance. Especially as the sensor elements based on graphene/PHBV are designed to work in wide temperature ranges including conditions below and above room temperature; therefore, it is crucial to determine their mechanical parameters at these conditions. DMA is useful to determine the crucial effect of graphene structures and their distribution on the polymer molecular relaxations (intensity and temperature range) and the character of interfacial polymer-graphene region. The storage modulus (E′), loss modulus (E″) and mechanical loss coefficient (tan δ) versus temperature, at established heating rate, for pure PHBV/(R,S)-PHB and the composite films with raw and oxidized MWCNTs as well as multilayered GNPs are shown in Figure 8.

Table 2 summarizes the viscoelastic parameters of pure PHBV/(R,S)-PHB blend and the composites filled with various graphene fillers. The storage modulus is closely correlated with the stiffness of the polymer mixture and composites in the glassy and melting area. Depending on the temperature range, the changes in the storage modulus for composite systems varied. The highest E’ values in the glassy state for composite with raw MWCNTs and ox-MWCNTs used simultaneously with GNPs indicate the higher susceptibility of the network of filler particles in the polymer to deformation from external sources. This is also the results of the dispersion and distribution quality of the filler in the polymer network, and the adhesion at an interface between filler and polymer [20,37,38]. The composite with MWCNTs is characterized by the highest storage modulus in melted region, while E’ value in glassy region is 27% higher compared to the PHBV/(R,S)-PHB matrix and 14% lower compared to composite with 1% ox-MWCNTs and 1% GNPs. A considerable reduction in the storage modulus of the composite with ox-MWCNTs over the whole temperature range in relation to composite with raw MWCNTs and composites with GNPs can be explained based on the dispersion characteristics observed in the SEM photographs. The lowest E’ values for PHBV/(R,S)-PHB with GNPs in glassy and melted states compared to the pure matrix and other composites could be explained by the greater tendency of GNPs to agglomerate as a result of their higher aspect ratio and larger surface area, their non-uniform dispersion, or weaker adhesion in the PHBV/(R,S)-PHB co-continuous phase.

A single relaxation peak was observed in the loss modulus versus temperature curves, which could correspond to the PHB glass transition. Regarding the literature reports, T_g_ of pure PHB depends strongly on the processing method and can be observed as 14 °C for micro-compounding using melt mixing with subsequent compression molding [6] and 11 °C for the procedure of solution casting [39]. While a glass transition temperature of PHBV is located in the range of −5 to 20 °C depending on the HV (hydroxyvalerate) content of the copolymer [40]. The T_g_ of PHBV with 12% of hydroxyvalerate was reported to be 10 °C when taken as the loss modulus maximum and 17 °C when taken as the tanδ peak maximum [41]. On the other hand, the measurement technique also affects T_g_ values, so a difference in T_g_ determined by DSC and DMA is expected for example. The maximum of the loss modulus peak for the pure PHBV/(R,S)-PHB system is −23 °C (taken as inflection of the storage modulus −24.3 °C). Considering the PHBV/(R,S)-PHB composites studied, T_g_ determined using E” temperature dependence can be observed in the range of 6.5–13 °C, while T_g_ values obtained as the inflection point of E’ curves are in the comparable range between 5.5 °C and 14 °C. Thus, a significant increase in the T_g_ of composites is noted compared to the unfilled PHBV/(R,S)-PHB mixture. The increase in T_g_ for all nanocomposites can be associated with the constraint of the segmental movement of the poly(3-hydroxybutyrate) molecular fragments resulting from the presence of carbon nanoparticles. The effect of the highest increase in T_g_ for PHBV/(R,S)-PHB filled with raw MWCNTs may be additionally related to the interparticle entanglement of the nanotubes despite the de-agglomeration at the composite preparation stage. The lower T_g_ for the composite filled simultaneously with MWCNTs and GNPs than for the composite only with MWCNTs indicates the increased mobility of PHB chains and the sliding between crystallites in the surrounding amorphous regions [42,43]. The width at half of the E” peak to all composites is lower than those of the pure PHBV/(R,S)-PHB matrix, showing strongly bonded filler nanoparticles with the polymer phase as confirmed by SEM observations.

### 3.7. Electrical and Piezoresistive Characterization

**Figure 9:**
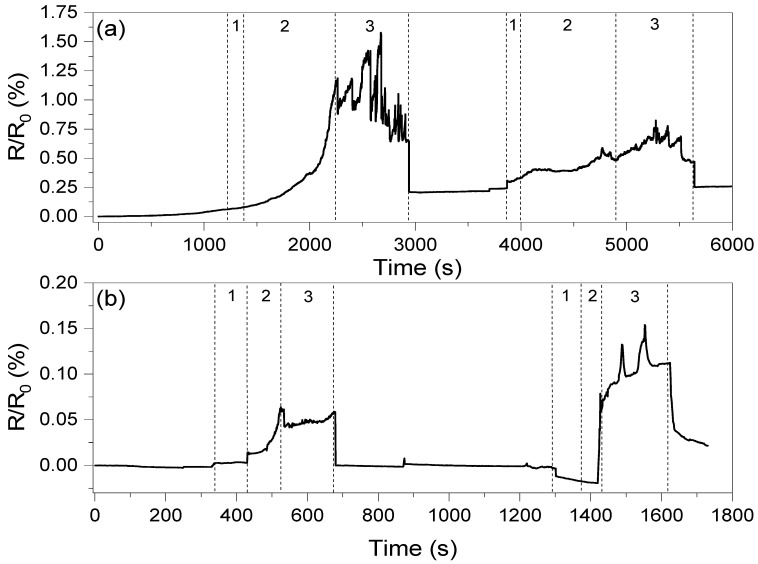
Resistance changes as a function of elongation registered for composites with (**a**) the ox-MWCNT/GNP mixture and (**b**) the MWCNT/GNP mixture. The numbers in the figure correspond to 1—deformation of composite, 2—stabilization of the composite resistance and 3—stable response level. The first deformation corresponds to ε = 1.5%, while the second corresponds to ε = 3.0%.

The conductivity values of the composites presented in Table 2 clearly show that the introduction of oxidized carbon nanotubes and GNP results in low conductivity of those systems. This is an effect of GNP agglomeration and high oxidation of CNT structures, respectively, as described in previous work [20]. On the other hand, the network formed by the carbon nanotubes resulted in only high conductivity, with very low potential for strain sensing, and therefore only hybrid systems with two graphene fillers were investigated in piezoresistive studies. The changes in resistance as a the function of deformation are shown in Figure 9.

The oxidized carbon nanotube system can be observed to show a higher response compared to the untreated CNT system. However, the system with the MWCNT/GNP hybrid filler shows one order of magnitude faster response and more stable response than the ox-MWCNT/GNP composite. The average response times for the PHBV/(R,S)-PHB +1%ox-MWCNTs+1%GNPs and PHBV/(R,S)-PHB +1%MWCNTs+1%GNPs composites are 1000 and 80 s, respectively. This is most likely because of the presence of –OH and –COOH groups on the surface of oxidized MWCNTs, which can result in better adhesion between filler particles and the polymer matrix, and therefore deformation of the conductive network is hindered. This resulted in a very long response time for the PHBV/(R,S)-PHB +1%ox-MWCNTs+1%GNPs composite. However, after release, the recovery time of both systems is similar, which may be a result of the high storage modulus at room temperature.

An observation should be made that a 1.5% deformation and recovery does not affect the conductivity of the PHBV/(R,S)-PHB +1%MWCNTs+1%GNP system, while for the system with oxidized carbon nanotubes, the resistance increases by approximately 0.2%. Furthermore, this deformation permanently affects the conductive network, causing a decrease in response at higher deformations, which was not observed for the biopolymer system with MWCNTs/GNPs. However, a deformation of 3% again affects the resistance, but in this case of both composites (by approximately 0.025).

## 4. Conclusions

Biopolymer composites of the PHBV/(R,S)-PHB blend (70:30 wt.%), including 2 wt.% of graphene nanofillers of different structures (GNPs and MWCNTs) used in various combinations, were each successfully fabricated with the solvent casting method. Piezoresistive sensors were subsequently prepared with the use of these composites.

- A study of the crystallinity degree of the polymer components in the PHBV/(R,S)-PHB blend, determined with the help of DSC and XRD methods, proved that the addition of (R,S)-PHB reduced the crystallinity of PHBV in the final mixture. Oppositely, the introduction of graphene fillers resulted in an increase in the PHBV crystallinity and a greater increase found in the composite containing 1% ox-CNTs+1%GNPs, and in the composite containing only GNPs.
- The porous morphology of the PHBV/(R,S)-PHB blend remains unchanged in the composites after the introduction of 2 wt.% graphene nanomaterials. Significant strengthening of the fracture surface was observed by AFM imaging for the graphene/biopolymer composites, which leads to a decrease in the crack number and an increase in the irregularity. TEM analysis showed a smaller size distribution of the spherical particle of PHBV/(R,S)-PHB in the composites compared to pure PHBV/(R,S)-PHB.
- The storage modulus representing the stiffness both in the glassy and melting regions decreased significantly when oxidized carbon nanotubes and GNP were used as individual fillers in the PHB/(R,S)-PHB blend. The highest increase in the storage modulus was found for the composite with ox-MWCNTs and GNPs demonstrating an effect of synergy in the case of oxidized MWCNTs and GNPs on the viscoelastic properties of the composite. The result also suggests the elastic susceptibility of the network of filler particles in the polymer to the deformation from the external sources and strong adhesion at the interface between the filler and polymer phases. The smaller values of the width at half of the peak of the loss modulus of all composites compared to the polymer blend confirms the strongly bonded filler to the polymer phases.
- The electrical conductivity of composites was observed to depend on the structure of graphene nanomaterials and their distribution in the PHBV/(R,S)-PHB matrix, as well as the interactions at the polymer–filler interface observed by SEM. The most promising piezoresistive responses were noted for composites with 1%ox-MWCNTs+1%GNPs and 1%MWCNTs+1%GNPs. However, a 1.5% deformation and recovery did not affect the conductivity of the PHBV/(R,S)-PHB +1%MWCNTs+1%GNP system, while for the system with oxidized carbon nanotubes, the resistance increases by approximately 0.2%. In addition, this deformation has a permanent effect on the conductive network, resulting in a decrease in the response of composites with ox-MWCNTs and GNPs at higher deformations, which was not observed for the biopolymer system with MWCNTs/GNPs.

## Figures and Tables

**Table 1:** Glass transition, melting parameters and crystallization degree of PHBV/(R,S)-PHB)-based composite systems.

Composite System	T_g_ [°C]	Melting				*X*_c_ [%] XRD
		*T*_m1_ [°C]	*T*_m2_ [°C]	Δ*H*_m_ [J/g]	*X*_cPHBV_ [%]	
PHBV film			176.9	92.8	64.0	64.0
PHBV/(R,S)-PHB	−6.5	132.2	144.9	27.7	27.1	34.9
+2% MWCNTs	0.3	140.6	160.9	45.6	45.9	40.5
+2% ox-MWCNTs	1.5	161.0	170.3	54.2	54.6	48.8
+2% GNPs	3.0	163.0	171.6	53.4	53.8	48.1
+1% MWCNTs+1%GNPs	3.3	164.1	171.9	53.6	54.0	48.6
+1% ox-MWCNTs+1%GNPs	3.6	163.9	173.4	36.3	37.1	39.1

**Table 2:** Dynamic mechanical parameters and conductivity for PHBV/(R,S)-PHB system and its composites with MWCNTs, ox-MWCNTs and GNPs.

Composite System	*E’*_−50°C_ [MPa]	*E’*_20°C_ [MPa]	*E’*_100°C_ [MPa]	*T_infE’_* [°C]	*T*_gE”_ [°C]	*E*”_max_ [MPa]	WH_E”_ [°C]	σ [S/cm]
PHBV/(R,S)-PHB	2999	727	109	−24.2	−23.0	252	18.6	-
+2%MWCNTs	3857	1360	285	14.4	13.3	418	15.3	1 × 10^−3^
+2%ox-MWCNTs	2489	705	136	7.8	9.0	290	14.6	1 × 10^−13^
+2%GNPs	1913	232	57	5.4	6.5	128	12.2	5 × 10^−13^
+1%MWCNTs+1%GNPs	3237	777	127	8.5	9.5	385	13.1	9 × 10^−5^
+1%ox-MWCNTs+1%GNPs	4505	1184	224	7.7	8.5	550	15.6	8 × 10^−6^

Annotation: *E’*_−50°C_, *E’*_20°C,_ *E’*_100°C_—the storage modulus at −50 °C, 20 °C and 100 °C, respectively; *T_infE’_*—the inflection temperature of the storage modulus curve; *T*_gE”_—the temperature of glass transition temperature at the maximum of the loss modulus; *E*”_max_—the loss modulus maximum value; WH_E”_—width at half of the loss modulus peak, σ—electrical conductivity.

## Data Availability

The original contributions presented in the study are included in the article, further inquiries can be directed to the corresponding authors.
